# Conflicting Role of Sarcopenia and Obesity in Male Patients with Chronic Obstructive Pulmonary Disease: Korean National Health and Nutrition Examination Survey

**DOI:** 10.1371/journal.pone.0110448

**Published:** 2014-10-29

**Authors:** Hyeon-Kyoung Koo, Joo-Hyun Park, Hye Kyeong Park, Hoon Jung, Sung-Soon Lee

**Affiliations:** Division of Pulmonary and Critical Care Medicine, Department of Internal Medicine, Ilsan Paik Hospital, Inje University College of Medicine, Goyang, Korea; University of Palermo, Italy

## Abstract

**Objective:**

To determine the impact of sarcopenia and obesity on pulmonary function and quality of life (QOL) in chronic obstructive pulmonary disease (COPD) patients.

**Research Design and Methods:**

Data were obtained from the Korea National Health and Nutrition Examination Survey, including data from health interviews, health examinations, nutritional questionnaires, and laboratory findings. Laboratory data included pulmonary function assessment and dual energy X-ray absorptiometry results. Sarcopenia was measured by dual energy X-ray absorptiometry, and obesity was defined by body mass index. Male COPD patients were then classified into 4 groups according to the presence of sarcopenia and obesity.

**Results:**

In male patients with COPD, the prevalence of sarcopenia was found to be 29.3%, and that of sarcopenic obesity was 14.2%. Furthermore, 22.5% of the patients observed in this study had impaired QOL. Following multivariable statistical analysis, both sarcopenia and obesity were independent risk factors for worsening lung function. Adjusted values of forced vital capacity and forced expiratory volume in 1 second were the lowest in the sarcopenic obesity group. Sarcopenia was also associated with more subjective activity limitation and poorer QOL; however obesity was related to less subjective limitation and better QOL after multivariable analysis. Adjusted value of QOL was the lowest in sarcopenic subjects without obesity, and the highest in obese subject without sarcopenia.

**Conclusions:**

Both sarcopenia and obesity were found to be associated with worsening lung function in male COPD patients. However, obesity was positively correlated with improved QOL while sarcopenia was negatively correlated with QQL.

## Introduction

Chronic obstructive pulmonary disease (COPD) is a heterogeneous disorder with a wide range of phenotypical variability and systemic manifestations [1]. Patients suffering from COPD are often described as either pink puffers or blue bloaters due to this heterogeneity. Inflammatory nature of this disease causes catabolic state, and leads them to generally wasted status [2]. Body mass index (BMI) is indicative of basic nutritional status and has previously been known to be predictor of mortality in COPD patients [3]–[6]. However, it is also reported that the value of fat free mass is better correlated with disease severity and physical performance than BMI in several studies including small number of COPD patients [7], [8].

The aim of this study was to classify COPD patients based on their muscle mass status and BMI, and verify their effects on physiological characteristics such as lung function, daily activities, and quality of life (QOL) in these patients.

## Materials and Methods

### Study population

The Korea National Health and Nutrition Examination Survey (KNHANES) is a series of cross-sectional and nationally representative population-based health and nutritional survey by the Korean Centers for Disease Control and Prevention. The KNHANES archives contain data collected since the first survey in 1998. KNHANES used a stratified multistage clustered probability sampling design, and the sampling units were based on geographical area, age, and sex. This survey consisted of a health interview, a health examination, and nutritional questionnaires. Pulmonary function test (PFT) was performed in subjects older than 40 years of age, and dual energy X-ray absorptiometry (DEXA; Discovery-WTM; Hologic Inc., Bedford, MA, USA) was performed for subjects older than 10 years of age. Skeletal muscle mass was measured by DEXA. In the current study, male COPD patients (≥40 years of age) between 2009 and 2011 survey were selected based on a PFT result of FEV_1_/FVC <0.7 and a history of smoking according to the Global Initiative for Chronic Obstructive Lung Disease (GOLD) guideline [1]. All the individuals in this survey participated voluntarily, and written informed consent was obtained from all participants by themselves. The survey protocol was approved by the institutional review board of the Korean Centers for Disease Control and Prevention.

### Pulmonary function test

A model 1022 Spirometer (SensorMedics; USA) was used for pulmonary function test. Spirometry was conducted with standardized equipment following guidelines from the American Thoracic Society/European Respiratory Society [9]. Spirometry was repeated at least three times to ensure reproducibility and validity. The PFT results were calculated based on the reference values from published predictive equations for Korean patient populations [10], using computer programs and reviewed by trained physicians.

### Definitions of sarcopenia and obesity

DEXA was performed to measure muscle mass, and the results of the DEXA were analyzed using industry standard techniques at the Korean Society of Osteoporosis with Hologic Discovery software (version 13.1) in its default configuration. Appendicular skeletal muscle mass (ASM) was measured as the sum of the lean soft tissue masses of the arms and legs by DEXA [11]. Skeletal muscle mass index (SMI) was calculated using ASM/weight (kg)×100, and sarcopenia was defined when SMI was less than 1 SD below the gender-specific mean for a young reference group between 20 and 39 years of age based on a previous Korean cohort study [12]–[14]. The cut-off value for sarcopenia in the reference groups was 30.8%, 29.8%, and 30.4% for the group from 2009, 2010, and 2011, respectively. The lowest value observed (2010) was set as the reference value for sarcopenia. Obesity was defined when subjects had a BMI greater than 25 kg/m^2^ based on the World Health Organization recommendations for Asian population-based classification [15]. The definition of central obesity followed the criteria for Asian individuals (waist circumference ≥90 cm for males).

### Activity limitation and impaired quality of life

Questionnaires used for data collection by KNHANES included assessments relative to feelings of subjective activity limitation, and included scale-based assessments for health-related QOL. Health-related QOL was measured using the validated Korean version of the 5-item self-administered EuroQOL instrument (EQ-5D). The EQ-5D is a generic questionnaire used to assess the QOL in patients with chronic disease [16], and reported to have a strong correlation with St Georges Respiratory Questionnaire (SGRQ) score in COPD patients [17]. The EQ-5D has a descriptive system and a visual analog scale (VAS), and it reports patients’ current health status. The descriptive assessments consisted of 5 items with 3 possible answers for each. The assessments covered the 5 dimensions relative to QOL: mobility; self-care; usual activities; pain/discomfort; and anxiety/depression. Each item can be used to represent profiles of health status or can be converted to a summary index (EQ5D index). The VAS is a measurement scale ranging from 0 (representative of the worst health status) to 100 (representative of the best health status). We also used 3 items that are used to grade dyspnea severity at MMRC scale (walking, bathing, and daily activities) [18] as part of the EQ-5D to assess ordinary activities of life. We defined limitation of ordinary activities based on descriptive difficulties in any one of these items.

### Statistical analysis

The data were analyzed with complex-sample analysis procedures using SAS version 9.3. We adjusted the analysis for the complex sample design of the survey using stratification, sampling weight variables, and clustering variables. In order to compare characteristics of each subgroup, general linear regression was used for continuous variables and logistic regression was used for categorical variables. Data were represented as mean ± standard error, or frequency (%). A *P*-value<0.005 was used to indicate statistical significance.

## Results

### Effects of sarcopenia and obesity on lung function

**Figure 1:**
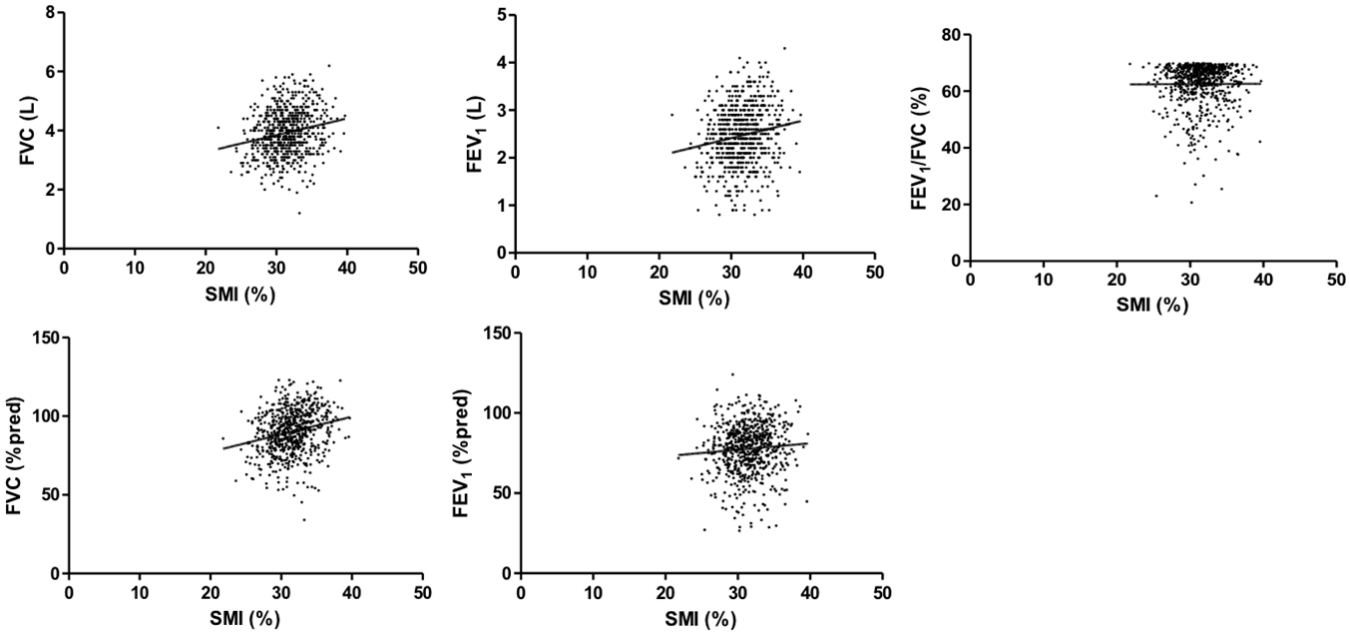
Correlation between muscle mass index and lung function. Legend: R^2^ for FVC (L), 0.048, *P*<0.001; FVC (% predicted), 0.068, *P*<0.001; FEV_1_ (L), 0.037, *P* = 0.001; FEV_1_ (% predicted), 0.007, *P* = 0.181, FEV_1_/FVC, 0.001, *P* = 0.670.

DEXA data were successfully retrieved for a total of 574 male adult patients with COPD. Sarcopenia, as assessed by complex-sample analysis, had a prevalence of 29.3%, and obesity was observed at a prevalence of 27.3% in Korean male COPD patients. The mean age of the COPD population was 64.0 (±0.6) years, and 103 (17.9%) individuals were never or previous light smokers, 231 (40.2%) were former smokers, and 240 (41.8%) were current smokers. The proportion of GOLD 1 COPD was 46.3%; GOLD 2 COPD was 48.6%, and GOLD 3–4 COPD was 5.1%. The proportion of GOLD 1 COPD was 46.3%; GOLD 2 was 48.6%; and GOLD 3–4 was 5.1%. The prevalence of sarcopenia in each GOLD group was 22.7%, 35.4%, and 30.9%, and that of obesity was 25.4%, 30.3%, and 16.9%, respectively. Due to the similar prevalence of sarcopenia, GOLD 2 and GOLD 3–4 groups were combined and redefined as the advanced COPD group. The association between muscle mass index and lung function is presented in Figure 1. The SMI was positively correlated with FVC (L), FVC (% predicted), and FEV_1_ (L). The comparison of demographics and clinical characteristics, including pulmonary functions according to the sarcopenic status, are summarized in Table 1, and laboratory findings are summarized in Table S1 in File S1. The baseline characteristics according to degree of airflow limitation are arranged in Table S2 at File S1. The mean age of patients in the sarcopenia group was 67.2±1.1 years, who were older than patients in the non-sarcopenic group (62.6±0.7 years). In addition, the sarcopenic group exhibited lower FVC (3.70±0.08 vs. 4.04±0.05, *P*<0.001) (L), FVC % predicted (84.9±1.2 vs. 92.4±0.8, *P*<0.001), and FEV_1_ (2.33±0.05 vs. 2.56±0.04, *P* = 0.001) (L). However, there was no significant difference in FEV_1_ (% predicted) and FEV_1_/FVC (%).

**Table 1:** Clinical characteristics according to sarcopenia and obesity status.

	Sarcopenia (−)			Sarcopenia (+)			*P* *
	Total	Obesity (−)	Obesity (+)	Total	Obesity (−)	Obesity (+)	
Number	419	328	91	155	82	73	
Age, years, mean	62.6±0.7	63.7±0.8	57.8±1.1	67.2±1.1	68.3±1.8	66.0±1.2	<0.001
Height, cm	167.2±0.4	166.9±0.45	168.6±0.9	166.7±0.6	166.4±0.8	166.9±0.9	0.46
Weight, kg	64.0±0.58	61.3±0.5	75.8±1.1	69.3±0.9	63.5±0.8	75.4±0.9	<0.001
BMI, kg/m^2^	22.8±0.2	22.0±0.1	26.7±0.2	24.9±0.3	22.9±0.2	27.0±0.2	<0.001
WC, cm	82.9±0.5	81.0±0.5	91.2±0.8	90.7±0.7	86.5±0.7	95.0±0.8	<0.001
SMI, %	32.8±0.1	32.9±0.2	32.0±0.2	28.2±0.1	28.3±0.2	28.0±0.2	<0.001
HTN, %	48.9±3.1	49.2±3.4	47.4±6.3	63.8±4.6	61.8±6.6	65.9±6.7	0.01
DM, %	17.4±2.5	18.0±2.9	15.3±4.4	29.5±4.6	28.8±6.2	30.2±6.8	0.02
Dyslipid, %	54.1±3.2	52.1±3.7	62.7±6.4	60.7±5.5	60.8±7.3	60.5±7.8	0.30
MI or HF, %	4.6±1.7	4.4±1.7	5.6±4.8	3.8±1.8	5.0±2.9	2.6±2.0	0.73
CKD, %	0.2±0.2	0.2±0.2	0	1.5±0.9	1.3±1.3	1.7±1.3	0.06
Smoking							
Current, %	49.9±2.9	50.3±3.3	48.2±6.2	32.3±4.9	32.1±6.7	32.5±6.5	0.01
Amount, PY	30.5±1.3	29.8±1.5	33.6±3.8	31.0±2.4	32.0±4.1	30.0±2.8	0.87
COPD stage, %							0.02
GOLD 1	50.6±3.1	50.0±3.5	53.7±6.3	35.9±4.5	38.5±6.8	33.2±6.4	
GOLD 2	44.4±3.1	44.0±3.6	45.6±6.3	58.7±4.5	56.2±6.9	61.4±6.7	
GOLD 3–4	5.0±1.4	5.9±1.7	0.7±0.7	5.4±2.1	5.4±3.0	5.4±3.0	
PFT							
FVC, L	4.04±0.05	3.97±0.05	4.35±0.10	3.70±0.08	3.68±0.10	3.72±0.11	<0.001
FVC, %predict	92.4±0.8	92.1±0.9	93.9±1.6	84.9±1.2	86.3±1.7	83.4±1.7	<0.001
FEV_1_, L	2.56±0.04	2.49±0.04	2.84±0.08	2.33±0.05	2.31±0.08	2.35±0.07	0.001
FEV_1_, %predict	78.9±0.9	78.2±1.1	82.1±1.8	76.2±1.3	77.4±2.1	75.0±1.6	0.01
FEV_1_/FVC, %	62.9±0.5	62.4±0.6	65.2±0.8	62.8±0.6	62.5±0.9	63.1±0.8	0.82

* P values were analyzed for patients with and without sarcopenia.

Abbreviation: BMI, body mass index; WC, waist circumference; SMI, skeletal muscle index; HTN, hypertension; DM, diabetes; Dyslipid, dyslipidemia; MI, myocardial infarction; HF, heart failure; CKD, chronic kidney disease; PY, pack-year; FVC, forced vital capacity; FEV_1_, forced expiratory volume in 1 second.

Since the sarcopenia group had significantly higher BMI, we further stratified the baseline characteristics according to the status of obesity as follows: absence of sarcopenia and obesity (S−O−); absence of sarcopenia and presence of obesity (S−O+); presence of sarcopenia and absence of obesity (S+O−); and presence of both sarcopenia and obesity (S+O+). The prevalence of S−O− was 57.6%; S−O+, was 13.1%; S+O−, was 15.1%; and S+O+, was 14.2%. The detailed clinical characteristics of 4 phenotypes are described in Table 1.

**Figure 2:**
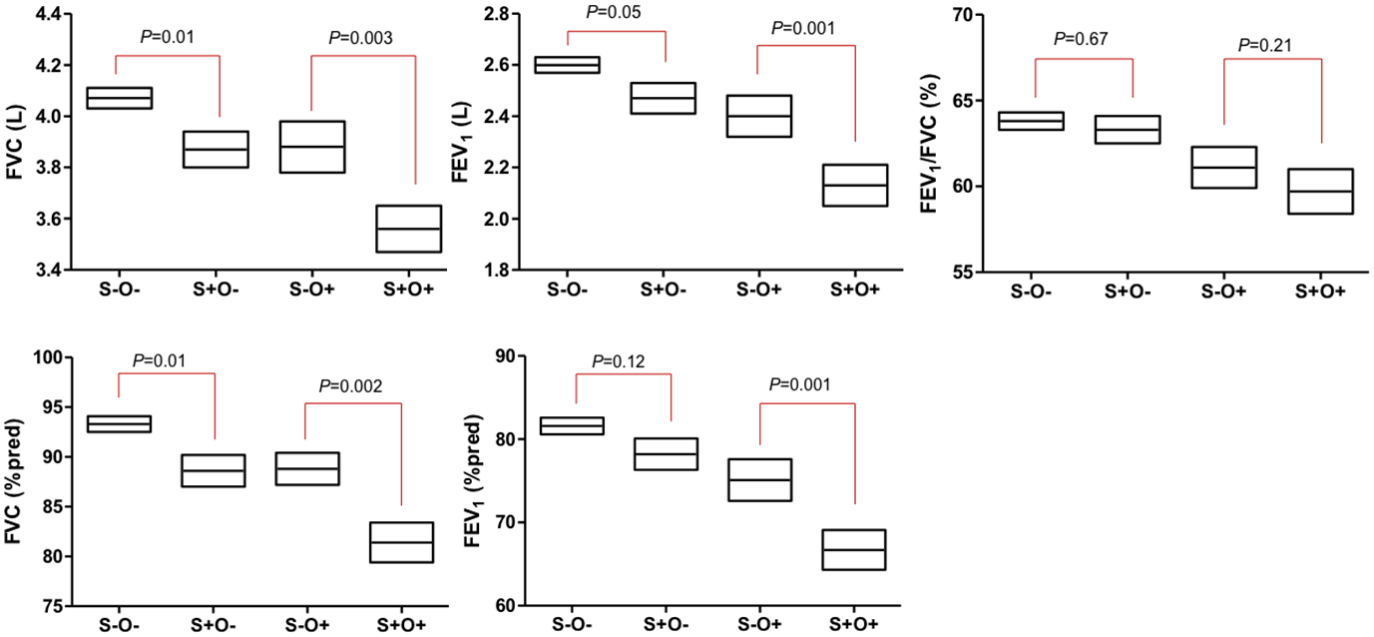
Comparison of lung function according to the presence of sarcopenia and obesity (mean, 95% CI). Legend: Values were adjusted by age, height, weight, obesity, and current smoking status. Abbreviation: absence of sarcopenia and obesity, S−O−; absence of sarcopenia and presence of obesity, S−O+; presence of sarcopenia and absence of obesity, S+O−; presence of both sarcopenia and obesity, S+O+.

Age, height, weight, and current smoking status were adjusted for lung function in addition to sarcopenia and obesity in multivariable analysis using general linear regression. Sarcopenia and obesity were independently associated with worse FVC (L), FVC (% predicted), FEV_1_ (L), and FEV_1_ (% predicted). The adjusted FEV_1_/FVC ratio was lower in obesity group, but was not statistically different in sarcopenia group (Table S3 in File S1). As there was no significant interaction between sarcopenia and obesity for lung function (FVC (L), *P* = 0.36; FVC (% predicted), *P* = 0.36; FEV_1_ (L), *P* = 0.20; and FEV_1_ (% predicted), *P* = 0.13), pulmonary function adjusted by age, height, weight, and current smoking status was compared between the 4 group classified based on the presence of sarcopenia or obesity. All adjusted values of lung function were observed to be significantly worse among patients with sarcopenic obesity except for FEV_1_/FVC ratio (Figure 2). As the sarcopenia group also had larger waist circumference, we divide and reclassified patients into 4 groups according to presence of sarcopenia and central obesity. However unlike obesity, central obesity was not associated with poorer lung function in multivariable analysis (Table S4 in File S1).

To evaluate the factors associated with advanced COPD (GOLD 2–4), variables including age, height, weight, smoking status, sarcopenia, and obesity were adjusted using logistic regression. Age, weight, obesity, and sarcopenia were independent risk factors associated with advanced COPD (model 1 in Table 2). Following multivariate analysis that included FVC (L) in addition to variables of model 1 (model 2 in Table 2), sarcopenia lost significance while age, height, weight, obesity, current smoking status, and FVC (L) were all significant.

**Table 2:** Factors associated with advanced COPD (% FEV_1_<80).

	GOLD 2–4 (N = 305)	GOLD 1 (N = 267)	*P*	Adjusted OR (95% CI)		
				Model 1	Model 2	
Age,mean±SD	63.2±0.7	64.8±0.9	0.204	1.03 (1.00–1.06)*	1.18 (1.11–1.26)*	
Height (cm)	167.0±0.4	167.1±0.5	0.909	0.95 (0.90–1.01)	0.73 (0.65–0.81)*	
Weight (kg)	65.0±0.7	66.1±0.7	0.275	1.09 (1.03–1.14)*	1.07 (1.01–1.13)*	
Obesity	29.0±3.2	25.4±3.2	0.436	2.56 (1.19–5.50)*	2.48 (1.01–6.13)*	
Sarcopenia	35.0±3.5	22.7±3.1	0.005	2.54 (1.57–4.09)*	1.61 (0.81–3.19)	
Curr smk (%)	46.4±3.5	42.6±3.6	0.417	1.03 (0.67–1.58)	2.07 (1.08–3.98)*	
Smk amt (PY)	30.5±1.6	30.8±1.8	0.946	―	―	
PFT						
FVC (L)	3.61±0.05	4.32±0.06	<0.001	―	151 (32.6–697)*	
FVC (%)	82.7±0.8	98.9±0.8	<0.001	―	―	
FEV_1_ (L)	2.17±0.04	2.86±0.04	<0.001	―	―	
FEV_1_ (%)	67.5±0.8	90.4±0.5	<0.001	―	―	
FEV_1_/FVC (%)	60.1±0.6	66.1±0.2	<0.001	―	―	

*indicates significance.

**Model 1**: variables include age, height, weight, obesity, sarcopenia, and smoking status.

**Model 2**: variables include FVC (L) in addition to variables included in Model 1.

FEV_1_ (% predicted) was not included in the multivariable analysis due to the risk of co-linearity.

Abbreviation: Curr smk, current smoker; Smk amt, smoking amount; PY, pack-year; FVC, forced vital capacity; FEV_1_, forced expiratory volume in 1 second.

### Impact of sarcopenia on activity limitation and quality of life

A total of 99 patients (representing a prevalence of 15.0%) reported subjective activity limitations and 127 patients (representing a prevalence of 22.5%) had impaired QOL, such as walking, bathing, and other daily activities. Prevalence of impairment of mobility, personal care, and daily activity was observed to be 18.1%, 4.2%, and 8.9%, respectively.

Older age, sarcopenia, and lower FVC (% predicted), FEV_1_ (L), and FEV_1_/FVC (%) were all associated with subjective activity limitation. However, obesity was observed to be correlated with better subjective activities in univariable and multivariable analysis (Table 3).

**Table 3:** Multivariate analysis for factors contributing to subjective exercise limitation.

	Subjective limitation (+)	Subjective limitation (−)	*P*	Adjusted OR	*P*
N	99	475		―	
Age, year, mean	68.0±1.4	63.3±0.6	0.001	1.05 (1.00–1.09)	0.05
Height. Cm	165.7±0.8	167.2±0.4	0.07	0.88 (0.78–0.98)	0.03
Weight, kg	63.6±1.1	65.8±0.6	0.09	―	
WC. Cm	86.1±0.9	85.0±0.5	0.32	―	
BMI. kg/m^2^	23.1±0.3	23.5±0.2	0.23	―	
**Obese, N (%)**	**20 (18.2%)**	**142 (28.6%)**	**0.07**	**0.40 (0.17–0.95)**	**0.04**
SMI, %	30.6±0.3	31.6±0.2	0.005	―	
**Sarcopenia, N (%)**	**30 (42.3%)**	**125 (27.2%)**	**0.02**	**2.10 (1.01–4.37)**	**0.04**
Current smoker, N (%)	32 (32.6%)	208 (46.8%)	0.26	―	
Smoking amount, PY	33.0±4.3	30.3±1.2	0.55	―	
Hb, g/dL	14.7±0.2	15.0±0.1	0.07	0.90 (0.69–1.17)	0.43
PFT					
FVC, L	3.64±0.11	3.98±0.04	0.003	―	
FVC, % predicted	86.0±1.9	90.8±0.7	0.018	0.90 (0.84–0.98)	0.03
FEV_1_, L	2.23±0.09	2.53±0.03	0.001	29.3 (1.4–630)	0.03
FEV_1_, % predicted	74.5±2.5	78.7±0.8	0.11	―	
FEV_1_/FVC, %	60.6±1.1	63.3±0.4	0.02	0.86 (0.76–0.97)	0.01
Ordinary limit	62 (66.4%)	65 (14.8%)	<0.001		
QOL					
EQ-5D index	0.77±0.02	0.96±0.01	<0.001	―	
EQ-VAS score	61.7±2.5	74.6±1.1	<0.001	―	

Abbreviation: BMI, body mass index; WC, waist circumference; SMI, skeletal muscle mass index; PY, pack-year; Hb, hemoglobin; FVC, forced vital capacity; FEV_1_, forced expiratory volume in 1 second; QOL, quality of life; EQ, EuroQOL instrument.

Weekly frequencies of exercise and exercise time categorized by intensity level were compared between the 4 groups. This information was collected using a patient-reported questionnaire, not accelerometers. Although was tendency for lower activity level in the sarcopenic nonobese group, there was no statistical difference between 4 groups, which may be due to high variance. A comparison of the 4 groups in terms of detailed exercise intensity and exercise amount is described in Table 4. Regarding the ordinary activities of life, neither sarcopenia nor lung function was a significant factor influencing difficulties, but only age showed significant association in multivariate analysis (Table S5 in File S1).

**Table 4:** Physical activity and activity limitation comparison between sarcopenia and obesity.

	S+O−	S+O+	S−O−	S−O+	Total	*P*
N	82	73	328	91	574	
Subjective limit	21 (31.5%)	9 (11.0%)	58 (12.9%)	11 (9.0%)	99 (15.0%)	0.03*
Ordinary limit	26 (36.7%)	13 (23.7%)	73 (20.5%)	15 (14.0%)	127 (22.5%)	0.01*
Weekly exercise level						
High act (/wk)						
0–2	74 (90.2%)	61 (75.6%)	273 (84.9%)	71 (80.9%)	481 (83.8%)	0.22
3–7	8 (9.8%)	12 (24.4%)	55 (15.2%)	18 (19.1%)	93 (16.2%)	
Times (h/wk)	7.8±1.9	8.6±1.5	13.1±4.4	9.7±1.6	11.4±2.6	0.71
Mod act (/wk)						
0–2	72 (88.4%)	54 (69.8%)	246 (75.8%)	60 (69.6%)	434 (76.1%)	0.13
3–7	10 (11.6%)	19 (30.3%)	82 (24.3%)	29 (24.3%)	140 (23.9%)	
Times (h/wk)	6.2±1.8	10.6±2.1	13.2±1.8	12.2±1.9	12.0±1.2	0.05
Walk act (/wk)						
0–2	21 (22.2%)	21 (31.7%)	104 (31.5%)	24 (28.5%)	170 (29.7%)	0.57
3–7	61 (77.9%)	52 (68.3%)	224 (68.5%)	65 (71.5%)	404 (70.3%)	
Times (h/wk)	8.0±0.8	9.4±1.6	10.8±1.4	9.8±1.2	10.0±0.9	0.29

* Indicate significance.

Abbreviation: absence of sarcopenia and obesity, S−O−; absence of sarcopenia and presence of obesity, S−O+; presence of sarcopenia and absence of obesity, S+O−; presence of both sarcopenia and obesity, S+O+;

**Figure 3:**
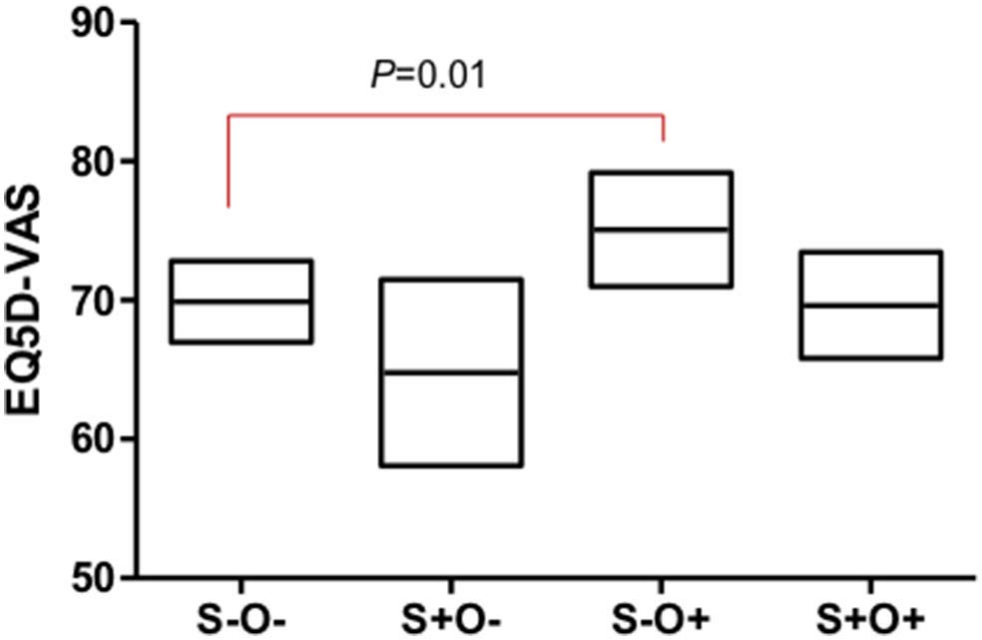
Comparison of quality of life according to the presence of sarcopenia and obesity (mean, 95% CI). Legend: Values were adjusted by age, COPD stage, sarcopenia, obesity, and current smoking status. Abbreviation: absence of sarcopenia and obesity, S−O−; absence of sarcopenia and presence of obesity, S−O+; presence of sarcopenia and absence of obesity, S+O−; presence of both sarcopenia and obesity, S+O+.

N, number; limit, limitation; act, activity; wk, week;

It is well known that advanced age and severity of COPD are related to impairment of health-related QOL [19], [20]. In our population, EQ5D index was 0.93 (±0.01), 0.94 (±0.01), and 0.86 (±0.04) (*P* = 0.14), while EQ5D-VAS was 73.2 (±1.6), 73.2 (±1.4), and 62.3 (±3.2) (*P* = 0.01) in the GOLD 1, 2, 3–4 COPD groups, respectively. Appendicular skeletal muscle mass index (SMI: percentage of ASM/weight) showed significant association in the simple regression with the EQ5D index (R^2^ = 0.027, *P* = 0.002). Therefore, we included variables such as age, stage of COPD, sarcopenia, obesity, and current smoking status with multivariable analysis for the EQ-5D index and EQ-5D VAS. The EQ-5D index was only associated with sarcopenia (0.89±0.02 vs. 0.93±0.01, *P* = 0.03). However, EQ5D-VAS was independently related to age (*P* = 0.04), stage of COPD (73.0±1.5 vs. 73.1±1.5 vs. 63.3±3.2, *P* = 0.02), sarcopenia (67.2±2.0 vs. 72.4±1.5, *P* = 0.03), and obesity (72.3±1.6 vs. 67.3±1.6, *P* = 0.01). There was no statistically significant interaction between sarcopenia and obesity for QOL (*P* = 0.94), and the adjusted value of each EQ5D-VAS index for S−O−, S−O+, S+O−, and S+O+ was 69.9 (±1.5), 75.1 (±2.1), 64.8 (±3.4), 69.6 (±1.9), respectively (Figure 3).

Though sarcopenia and obesity are known to be related to metabolic syndrome (Table 1) [21], [22], presence of hypertension (*P* = 0.49), diabetes (*P* = 0.40), and dyslipidemia (*P* = 0.15) had no effect on the values of adjusted EQ5D index or EQ5D-VAS score, and no interaction with sarcopenia or obesity in COPD patients. Sarcopenia and obesity were independently associated with QOL index (*P* = 0.02) despite adding these variables of metabolic syndrome to a previous multivariable analysis (age, *P* = 0.01; stage of COPD, *P* = 0.08; current smoking status, *P* = 0.02; hypertension, *P* = 0.68; diabetes, *P* = 0.40; and dyslipidemia, *P* = 0.18). When further adding the history of myocardial infarction (MI) or heart failure (HF) and chronic kidney disease (CKD) as covariates, multivariate analysis also revealed the independent association of sarcopenia and obesity with the EQ5D-VAS score (age, *P* = 0.02; stage of COPD, *P* = 0.06; current smoking status, *P* = 0.04; hypertension, *P* = 0.53; diabetes, *P* = 0.38; dyslipidemia, *P* = 0.15; MI or HF, *P* = 0.27; and CKD, *P* = 0.53). Even though we stratified into 4 groups according to the presence of sarcopenia and central obesity, abdominal obesity was not a significant factor contributing to a worse EQ-5D VAS score (absence of sarcopenia and central obesity, 70.2±1.5; absence of sarcopenia and presence of central obesity, 70.9±2.6; presence of sarcopenia and absence of central obesity, 72.1±2.5; presence of sarcopenia and obesity, 63.5±2.9; *P* = 0.11).

## Discussion

Our results show that nearly 30% of patients were found to be sarcopenic, and half of them were also found to be obese in Korean male COPD population. Sarcopenia and obesity were revealed as independent risk factors for worse lung function and advanced COPD. Lung function was found to be the lowest in the sarcopenic obesity group. Nearly a quarter of patients from the current study exhibited impaired daily activities. Although actual exercise time was not different and ordinary activities were not affected by sarcopenia or obesity after multivariable analysis, sarcopenic groups felt more subjective activity limitation and worsened QOL. Interestingly, the obesity group exhibited less subjective limitation and better QOL. Lung function of the non-sarcopenic obesity (S−O+) group and the non-obese sarcopenic (S+O−) groups were found to be similar; however, QOL was observed to be the highest among the non-sarcopenic obesity (S−O+) group, while the non-obese sarcopenic (S+O−) group exhibited the lowest QOL. Prevalence of sarcopenia increased from GOLD 2 COPD; but QOL was not different between the GOLD 1 and GOLD 2 COPD groups, and it decreased significantly from the GOLD 3 COPD group.

Sarcopenia and obesity both influence the severity of COPD. Following multivariable analysis for advanced COPD, both sarcopenia and obesity were independently associated in model 1, but sarcopenia lost significance after FVC (L) was added in model 2. Influence of sarcopenia on the severity of COPD, which is classified by FEV_1_ (% predicted), would be contributable to a decline in FVC. On the contrary, obesity can affect FVC but it more influenced the decline in FEV_1._ This may explain the phenotypes of sarcopenic pink-puffers with combined obstructive/restrictive pattern of lung function, and obese blue-bloaters with predominantly obstructive pattern of lung function.

However, unlike sarcopenia, obesity acts as a protective factor for activity and QOL, independent of the negative effects on lung function. Thus, even though the sarcopenic obesity (S+O+) group had the worst lung function, the non-obese sarcopenic (S+O−) group felt the worst QOL. This discrepancy between lung function and QOL might explain why patients exhibit more symptoms despite good lung function (GOLD B), or exhibit fewer symptoms despite poor lung function (GOLD C) [1], and emphasizes the importance of evaluation for sarcopenia and obesity in COPD patients.

Our study had several strengths. First, this study revealed the comprehensive effect of sarcopenia and obesity in COPD patients. Previous studies compared the prognostic efficacies of BMI and sarcopenia [7], [8], but these parameters were found to have different roles in COPD pathophysiology. Even though there could be many hidden confounding variables that can account for these differences, this classification into 4 groups is simple and could be easily applied to clinical practice to aid in the prediction of patients’ characteristics. Second, to our knowledge, this is the largest study involving COPD patients that included muscle mass data, and the prevalence of sarcopenia could be retrieved owing to the unique study design. Furthermore, we used DEXA for muscle mass measurement, which is more precise than bioelectrical impendence analysis that had been frequently used in previous studies [3]. Screening for comorbidities such as osteoporosis is recommended under the current GOLD guidelines, and routine DEXA may support this function as well as be informative for sarcopenia diagnosis.

For the correct interpretation of the present results, the limitations of this study should also be noted. First, we did not evaluate post-bronchodilator FEV_1_ and FVC, which are more commonly used as lung function parameters in COPD patients [23], it was part of the national mass screening survey. Hence, we could not obtain data on the diffusing capacity for carbon monoxide (DLCO), emphysema status, coexistence of bronchiectasis, or variables reflecting functional status, such as the 6-minute walking distance, COPD assessment test (CAT score), St George’s Respiratory Questionnaire (SGRQ), handgrip muscle strength, and mid-thigh circumferences. Because the definition of sarcopenia based on SMI could be influenced by weight, further research using other criteria such as mid-thigh circumferences or handgrip muscle strength would verify the role of sarcopenia and obesity in COPD patients. In addition, we used EuroQOL as a QOL index, which is a generic questionnaire, not a disease-specific questionnaire such as Chronic Respiratory Disease Questionnaire (CRQ) or SGRQ. A significant amount of information was obtained using a patient-reported questionnaire, and data on weekly exercise frequencies and exercise amount was collected retrospectively. This subjective assessment is somewhat inaccurate owing to many biases, which could be the cause of the insignificancy of exercise level among the 4 groups. A form of objective measurement, such as an accelerometer could help us to better understand the correlation between exercise level, sarcopenia, and obesity. Furthermore, we could not completely differentiate COPD from asthma using the self-reported questionnaires because most patients had known their disease indiscriminately. Therefore, this could represent contamination by longstanding, smoking-induced asthma or overlap syndrome in our study population. Second, the number of women who fulfilled the inclusion criteria was small; hence, women were excluded from our study due to difficulties in calculation. Further analysis aimed at women would be needed near future. Third, we could not identify the possible mechanism for these results because this is a part of a cross-sectional study. Further detailed larger trials should be conducted to confirm our findings.

In conclusion, although both sarcopenia and obesity were related with worsened lung function, obesity was positively correlated to better QOL, which is contrary to the negative correlation observed with sarcopenia.

## Supporting Information

File S1 **Supplemental tables. Table S1.** Laboratory findings according to sarcopenia and obesity status. **Table S2.** Clinical characteristics according to degree of airflow limitation. **Table S3.** Multivariable analysis for sarcopenia and obesity influence on pulmonary function A) Sarcopenia. **Table S4.** Multivariate analysis for sarcopenia and central obesity influence on pulmonary function. **Table S5.** Multivariate analysis for factors contributing to limitation of ordinary activities.
(DOCX)
