# Supplementary material for: Conflicting Role of Sarcopenia and Obesity in Male Patients with Chronic Obstructive Pulmonary Disease: Korean National Health and Nutrition Examination Survey
Source: PLoS One. 2014 Oct 29;9(10):e110448. doi: 10.1371/journal.pone.0110448 (PMC4212941; doi:10.1371/journal.pone.0110448)
Supplement: File S1 — Supplemental tables. Table S1. Laboratory findings according to sarcopenia and obesity status. Table S2. Clinical characteristics according to degree of airflow limitation. Table S3. Multivariable analysis for sarcopenia and obesity influence on pulmonary function A) Sarcopenia. Table S4. Multivariate analysis for sarcopenia and central obesity influence on pulmonary function. Table S5. Multivariate analysis for factors contributing to limitation of ordinary activities. (DOCX) [file pone.0110448.s001.docx]

**Supplement**

Table S1. Laboratory findings according to sarcopenia and obesity status

|  | Sarcopenia(-) | | | Sarcopenia(+) | | | *P** |
| --- | --- | --- | --- | --- | --- | --- | --- |
|  | Total | Obesity(-) | Obesity(+) | Total | Obesity(-) | Obesity(+) |
| Total chol, mg/dL | 187.7 ± 2.8 | 188.0 ± 3.3 | 186.8 ± 4.7 | 181.5 ± 3.2 | 179.8 ± 4.8 | 183.3 ± 4.2 | 0.14 |
| HbA1c, % | 6.3 ± 0.1 | 6.4 ± 0.2 | 6.2 ± 0.2 | 6.6 ± 0.2 | 6.6 ± 0.2 | 6.7 ± 0.2 | 0.20 |
| Hb, g/dL | 14.9 ± 0.1 | 14.8 ± 0.1 | 15.5 ± 0.1 | 15.0 ± 0.1 | 14.8 ± 0.2 | 15.2 ± 0.2 | 0.67 |
| Vitamin D, ng/mL | 20.2 ± 0.5 | 20.4 ± 0.5 | 19.7 ± 0.7 | 18.8 ± 0.7 | 19.0 ± 1.2 | 18.6 ± 0.8 | 0.11 |
| FBS, mg/dL | 103.0 ± 1.3 | 102.4 ± 1.5 | 105.5 ± 3.0 | 106.6 ± 2.2 | 106.9 ± 3.2 | 106.2 ± 2.7 | 0.17 |

* *P* values were analyzed for patients with and without sarcopenia

Abbreviation: total chol, total cholesterol; HbA1c, hemoglobin A1c; Hb, hemoglobin; FBS, fasting blood sugar level

Table S2. Clinical characteristics according to degree of airflow limitation

|  | Total | GOLD 1 | GOLD 2 | GOLD 3-4 |
| --- | --- | --- | --- | --- |
| Age, years, mean | 64.0 ± 0.6 | 64.8 ± 0.9 | 63.0 ± 0.8 | 65.1 ± 2.0 |
| Height, cm | 167.0 ± 0.3 | 167.1 ± 0.5 | 167.0 ± 0.5 | 166.9 ± 1.3 |
| Weight, kg | 65.5 ± 0.5 | 66.1 ± 0.7 | 65.7 ± 0.8 | 58.5 ± 2.1 |
| BMI, kg/m^2^ | 23.4 ± 0.1 | 23.6 ± 0.2 | 23.5 ± 0.2 | 21.0 ± 0.8 |
| SMI, % | 31.4 ± 0.2 | 31.8 ± 0.2 | 31.0 ± 0.2 | 31.8 ± 1.0 |
| WC, cm | 85.2 ± 0.5 | 85.7 ± 0.6 | 85.2 ± 0.8 | 80.9 ± 2.3 |
| Smoking |  |  |  |  |
| Former, N (%) | 231 (40.2%) | 116 (43.4%) | 105 (38.2%) | 10 (33.3%) |
| Current, N (%) | 240 (41.8%) | 106 (39.7%) | 120 (43.6%) | 13 (4.7%) |
| Smoking amount (PY) | 30.7 ± 1.2 | 30.8 ± 1.8 | 30.3 ± 1.7 | 33.0 ± 3.6 |
| PFT |  |  |  |  |
| FEV_1_, L | 2.49 ± 0.03 | 2.86 ± 0.04 | 2.26 ± 0.03 | 1.32 ± 0.08 |
| FEV_1_, %predict | 78.1 ± 0.8 | 90.4 ± 0.5 | 70.2 ± 0.6 | 41.7 ± 1.6 |
| FVC, L | 3.94 ± 0.04 | 4.32 ± 0.06 | 3.68 ± 0.05 | 2.89 ± 0.15 |
| FVC, %predict | 90.2 ± 0.7 | 98.9 ± 0.8 | 84.3 ± 0.7 | 67.5 ± 2.8 |
| FEV_1_/FVC, % | 62.9 ± 0.4 | 66.1 ± 0.2 | 61.5 ± 0.5 | 46.6 ± 1.9 |

Abbreviation: BMI, body mass index; SMI, skeletal muscle index; WC, waist circumference; PY, pack-year; FVC, forced vital capacity; FEV_1_, forced expiratory volume in 1 second; %predict, % predicted

Table S3. Multivariable analysis for sarcopenia and obesity influence on pulmonary function

A) Sarcopenia

|  | Sarcopenia (+) | Sarcopenia (-) | *P* | Adjusted OR* | 95% CI |
| --- | --- | --- | --- | --- | --- |
| Age, year | 67.2 ± 1.1 | 62.6 ± 0.7 | <0.01 | ― | ― |
| Height, cm | 166.6 ± 0.6 | 167.2 ± 0.4 | 0.46 | ― | ― |
| Weight, kg | 69.3 ± 0.9 | 64.0 ± 0.6 | <0.01 | ― | ― |
| Current smk, % | 32.3 ± 4.9 | 49.9 ± 2.9 | 0.003 |  |  |
| FVC, L | 3.70 ± 0.08 | 4.04 ± 0.05 | <0.01 | 0.92 | 0.88-0.96 |
| FVC, %predict | 84.9 ± 1.2 | 92.4 ± 0.8 | <0.01 | 0.97 | 0.95-0.98 |
| FEV_1_, L | 2.33 ± 0.05 | 2.56 ± 0.04 | 0.001 | 0.91 | 0.87-0.97 |
| FEV_1_, %predict | 76.2 ± 1.3 | 78.9 ± 0.9 | 0.10 | 0.98 | 0.96-0.99 |
| FEV_1_/FVC, % | 62.8 ± 0.6 | 62.9 ± 0.5 | 0.82 | 0.98 | 0.95-1.02 |

* Adjusted by age, height, weight, current smoking status, and obesity (Adjusted odds ratios of age, height, weight or current smoking status in addition to each values of lung function are not shown)

Abbreviation: Current smk, current smoker; FVC, forced vital capacity; %predict, %predicted; FEV_1_, forced expiratory volume in 1 second

B) Obesity

|  | Obesity (+) | Obesity (-) | *P* | Adjusted OR* | 95% CI |
| --- | --- | --- | --- | --- | --- |
| Age, year | 62.1 ± 0.9 | 64.7 ± 0.7 | 0.03 | ― | ― |
| Height, cm | 167.7 ± 0.6 | 166.8 ± 0.4 | 0.20 | ― | ― |
| Weight, kg | 75,6 ± 9,7 | 61.8 ± 0.5 | <0.01 | ― | ― |
| Current smk, % | 39.9 ± 4.6 | 46.5 ± 3.0 | 0.22 |  |  |
| FVC, L | 4.02 ± 0.08 | 3.91 ± 0.05 | 0.24 | 0.43 | 0.34-0.55 |
| FVC, %predict | 88.4 ± 1.3 | 90.9 ± 0.8 | 0.11 | 0.63 | 0.56-0.72 |
| FEV_1_, L | 2.58 ± 0.06 | 2.45 ± 0.04 | 0.07 | 0.14 | 0.08-0.27 |
| FEV_1_, %predict | 78.4 ± 1.2 | 78.0 ± 1.0 | 0.81 | 0.52 | 0.45-0.61 |
| FEV_1_/FVC, % | 64.1 ± 0.6 | 62.4 ± 0.5 | 0.02 | 0.43 | 0.35-0.54 |

* Adjusted by age, height, weight, current smoking status, and sarcopenia (Adjusted odds ratios of age, height, weight or current smoking status in addition to each values of lung function are not shown)

Abbreviation: Current smk, current smoker; FVC, forced vital capacity; %predict, %predicted; FEV_1_, forced expiratory volume in 1 second

Table S4. Multivariate analysis for sarcopenia and central obesity influence on pulmonary function

A) Sarcopenia

|  | Sarcopenia (+) | Sarcopenia (-) | *P* | Adjusted OR* | 95% CI |
| --- | --- | --- | --- | --- | --- |
| Age, year | 67.2 ± 1.1 | 62.6 ± 0.7 | <0.01 | ― | ― |
| Height, cm | 166.6 ± 0.6 | 167.2 ± 0.4 | 0.46 | ― | ― |
| Weight, kg | 69.3 ± 0.9 | 64.0 ± 0.6 | <0.01 | ― | ― |
| Current smk, % | 32.3 ± 4.9 | 49.9 ± 2.9 | 0.003 |  |  |
| FVC, L | 3.70 ± 0.08 | 4.04 ± 0.05 | <0.01 | 0.93 | 0.89-0.97 |
| FVC, %predict | 84.9 ± 1.2 | 92.4 ± 0.8 | <0.01 | 0.97 | 0.95-0.99 |
| FEV_1_, L | 2.33 ± 0.05 | 2.56 ± 0.04 | 0.001 | 0.92 | 0.87-0.97 |
| FEV_1_, %predict | 76.2 ± 1.3 | 78.9 ± 0.9 | 0.10 | 0.98 | 0.96-0.99 |
| FEV_1_/FVC, % | 62.8 ± 0.6 | 62.9 ± 0.5 | 0.82 | 0.98 | 0.95-1.02 |

* Adjusted by age, height, weight, current smoking status, and central obesity (Adjusted odds ratios of age, height, weight or current smoking status in addition to each values of lung function are not shown)

Abbreviation: Current smk, current smoker; FVC, forced vital capacity; %predict, %predicted; FEV_1_, forced expiratory volume in 1 second

B) Central obesity

|  | Abdominal obesity(+) | Abdominal obesity(-) | *P* | Adjusted OR* | 95% CI |
| --- | --- | --- | --- | --- | --- |
| Age, year | 62.1 ± 0.9 | 64.7 ± 0.7 | 0.16 | ― | ― |
| Height, cm | 167.7 ± 0.6 | 166.8 ± 0.4 | <0.01 | ― | ― |
| Weight, kg | 75.6 ± 0.7 | 61.8 ± 0.4 | <0.01 | ― | ― |
| Current smk, % | 34.3 ± 4.6 | 48.6 ± 3.0 | 0.01 |  |  |
| FVC, L | 3.98 ± 0.09 | 3.91 ± 0.05 | 0.47 | 0.99 | 0.93-1.06 |
| FVC, %predict | 87.9 ± 1.4 | 91.2 ± 0.8 | 0.05 | 1 | 0.97-1.03 |
| FEV_1_, L | 2.54 ± 0.06 | 2.46 ± 0.04 | 0.28 | 0.98 | 0.91-1.07 |
| FEV_1_, %predict | 79.1 ± 1.3 | 77.7 ± 1.03 | 0.41 | 1 | 0.97-1.02 |
| FEV_1_/FVC, % | 63.6 ± 0.5 | 62.5 ± 0.5 | 0.12 | 0.99 | 0.93-1.05 |

* Adjusted by age, height, weight, current smoking status, and sarcopenia (Adjusted odds ratios of age, height, weight, current smoking status in addition to each values of lung function are not shown)

Abbreviation: Current smk, current smoker; FVC, forced vital capacity; %predict, %predicted; FEV_1_, forced expiratory volume in 1 second

Table S5. Multivariate analysis for factors contributing to limitation of ordinary activities

|  | Ordinary activity limitation(+) | Ordinary activity limitation(-) | *P* | Adjusted OR | *P* |
| --- | --- | --- | --- | --- | --- |
| N | 127 | 447 |  | ― |  |
| Age, year, mean | 68.4 ± 0.95 | 62.8 ± 0.59 | <0.001 | 1.04 (1.00, 1.08) | 0.02 |
| Height. cm | 165.7 ± 0.6 | 167.4 ± 0.4 | 0.04 | 0.98 (0.93, 1.04) | 0.56 |
| Weight, kg | 63.9 ± 0.7 | 65.9 ± 0.6 | 0.09 | ― |  |
| WC, cm | 85.1 ± 0.5 | 85.5 ± 0.8 | 0.78 | ― |  |
| BMI, kg/m^2^ | 23.2 ± 0.2 | 23.5 ± 0.2 | 0.43 | ― |  |
| Obese, N (%) | 28 (22.9%) | 134 (28.2%) | 0.07 | 0.75 (0.40, 1.39) | 0.35 |
| SMI, % | 30.6 ± 0.2 | 31.7 ± 0.1 | 0.004 | ― |  |
| Sarcopenia, N % | 39 (39.7%) | 116 (26.4%) | 0.02 | 1.62(0.84, 3.11) | 0.15 |
| Current smoker, N % | 46 (35.0%) | 194 (47.5%) | 0.52 | ― |  |
| Smoking amount, PY | 32.3 ± 2.6 | 30.2 ± 1.2 | 0.55 | ― |  |
| Hb, g/dL | 14.6 ± 0.1 | 15.0 ± 0.1 | 0.01 | 0.89 (0.72, 1.09) | 0.26 |
| PFT |  |  |  |  |  |
| FVC, L | 3.68 ± 0.08 | 4.00 ± 0.04 | 0.001 | ― |  |
| FVC, %pred | 87.1 ± 1.4 | 91.0 ± 0.7 | 0.03 | 0.99 (0.96, 1.02) | 0.59 |
| FEV_1_, L | 2.30 ± 0.06 | 2.54 ± 0.03 | 0.002 | 0.99 (0.42, 2.36) | 0.99 |
| FEV_1_, %pred | 77.3 ± 1.7 | 78.3 ± 0.8 | 0.64 | ― |  |
| FEV_1_/FVC, % | 62.1 ± 0.7 | 63.1 ± 0.4 | 0.22 | ― |  |
| QOL |  |  |  |  |  |
| EQ-5D index | 0.75 ± 0.01 | 0.99 ± 0.01 | <0.001 | ― |  |
| EQ-VAS score | 63.8 ± 2.7 | 75.2 ± 1.0 | <0.001 | ― |  |

Abbreviation: N, number; WC, waist circumference; BMI, body mass index; SMI, smooth muscle index; PY, pack-year; Hb, hemoglobin; FVC, forced vital capacity; FEV_1_, forced expiratory volume in 1 second; QOL, quality of life
